# A cross-sectional study on Chinese oncology nurses’ knowledge of bone health among cancer patients

**DOI:** 10.1007/s00520-023-07966-2

**Published:** 2023-08-01

**Authors:** Jing Shan, Sumei Lv, Lu Chen, Tianhua Li, Jingwen Li, Shuangyan Wang, Congcong Zhang

**Affiliations:** 1 Department of Orthopedics, The Fourth Hospital of Hebei Medical University, Hebei Provincial Cancer Institute, No. 12, Jiankang Road, Chang’an District, Shijiazhuang, Hebei China; 2 Nursing Department, The Fourth Hospital of Hebei Medical University, Hebei Provincial Cancer Institute, Shijiazhuang, Hebei China; 3 Department of Gynecological, The Fourth Hospital of Hebei Medical University, Hebei Provincial Cancer Institute, Shijiazhuang, Hebei China

**Keywords:** Oncology nurse, Skeletal-related events, Cancer treatment–induced bone loss, Bone health, Status survey

## Abstract

**Objective:**

To understand the knowledge status, obstacle factors, and management confidence of oncology nurses on the bone health of cancer patients, and in addition to provide reference for establishing bone health knowledge training system for oncology nurses and guiding them to manage bone health of cancer patients.

**Methods:**

A total of 602 nurses engaged in oncology nursing in 6 hospitals in Hebei Province were selected by cluster sampling, and an online anonymous survey was conducted by sending questionnaires to oncology nurses from the Hebei Cancer Prevention and Control Association. The questionnaire was developed by the study team. There are 4 parts, namely general information, nurses’ role and job responsibilities, knowledge of skeletal-related events (SREs) and cancer treatment–induced bone loss (CTIBL), and understanding and confidence in bone health management, for a total of 33 questions.

**Results:**

Thirty-seven percent of oncology nurses received training on bone health and other related contents; 40.48% of oncology nurses used domestic and foreign guidelines when managing patients with bone metastases or CTIBL. Only approximately one-third of oncology nurses had confidence in managing the side effects of bone metastases and bone modification drugs and identifying patients at risk of CTIBL and fracture; only 33.04% of oncology nurses believed that weight-bearing exercise can prevent bone loss; less than 50% of oncology nurses believed that aromatase inhibitor therapy, ovarian suppression therapy, androgen deprivation therapy, and low body weight were risk factors for pathological fractures. The reasons that hindered oncology nurses from optimizing the management of patients with bone metastases and understanding the preventive measures and risk factors for bone loss mainly included lack of relevant knowledge training, lack of understanding of effective intervention measures, and lack of training and professionalism of specialized nurses, including insufficient development time and guidelines for clinical nursing practice.

**Conclusion:**

Managers must continuously improve the training system of oncology nurses, enrich the content of training pertaining to bone health for cancer patients, formulate clinical nursing practice guidelines, and give oncology nurses more time for professional development.

## Introduction

Among patients with advanced cancer, up to 89% of prostate cancer, 75% of breast cancer, and 40% of lung cancer patients will develop bone metastasis during the course of the disease [1]. Skeletal-related events refer to the sum of a series of bone complications caused by disease progression in patients with advanced cancer bone metastases, including bone pain (BP), pathological fracture (PF), spinal cord compression (SCC), tumor-induced hypercalcemia (TIH), and surgery or radiation therapy due to the above conditions [2]. The incidence of skeletal-related events (SREs) in patients with breast cancer, lung cancer, and prostate cancer was as high as 63%, 59%, and 52%, respectively, during initial diagnosis or follow-up [3]. Cancer treatment–induced bone loss (CTIBL) is a complication of antitumor therapy. Hormone therapy, radiotherapy, chemotherapy, and immunotherapy can lead to decreased bone mineral density, osteopenia, or osteoporosis [4, 5]. These factors have seriously affected the bone health of cancer patients, increased the risk of fractures, affected their quality of life, increased mortality, and brought considerable health and economic burdens to patients and the medical system [6, 7]. Therefore, early identification of SREs, screening of patients with a high risk of CTIBL, and taking preventive measures and health education are important to maintain bone health [4, 8]. Bone health can be promoted and maintained, and osteoporosis and pathological fractures can be prevented by bone-modifying drug therapy, adequate intake of calcium and vitamin D supplementation, weight-bearing exercise, and lifestyle changes [9, 10].

Bone health is an important aspect of cancer care, requiring special attention from the nursing team, with detailed assessment and guidance [8, 11]. Optimization of bone health for cancer patients requires an interdisciplinary approach including medical oncology, radiotherapy, orthopedics, pain, palliative care, and other fields, as well as professional nurses, pharmacists, and physical therapists [12]. Oncology nurses play an important promoting and supporting role in bone health. Improving oncology nurses’ ability to recognize, assess, and manage bone health can improve patients’ compliance with bone-modifying drug therapy and report its treatment effect and adverse reactions [13, 14], thereby delaying the occurrence of SREs and improving bone health outcomes and quality of life in cancer patients [15].

Research on bone health in cancer patients mainly focuses on the prediction, treatment, risk factors, clinical characteristics, economic analysis of SREs, and the pathogenesis, clinical significance, and drug treatment of CTIBL. Research on bone health care and support for cancer patients shows that cancer nurses are important members of the multidisciplinary management of bone health in cancer patients and play the roles of educators, observers, evaluators, and supporters in bone health management [16–18], assisting in diagnosis and providing therapeutic interventions. Patients with advanced cancer who develop SREs and CTIBL benefit from the support and care provided by oncology nurses, such as quality of life, disease-related knowledge, medication compliance, and lifestyle [19, 20]. However, there is still a lack of quantitative research on oncology nurses’ knowledge of cancer patients’ bone health. The purpose of this study is to understand the knowledge status, obstacle factors, and management confidence of oncology nurses on the bone health of cancer patients, and in addition to provide reference for establishing bone health knowledge training system for oncology nurses and guiding them to manage bone health of cancer patients.

## Methods

### Samples

A total of 602 oncology nurses (according to the sample size calculation formula for cluster sampling) were selected from 6 hospitals by cluster sampling. Inclusion criteria were as follows: in-service nurses with licensed nurse qualifications; engaged in solid tumor nursing. Exclusion criteria were as follows: nurses who were interns or advanced training; hematology oncology nurse. In this study, questionnaires were distributed to cancer nurses by the Hebei Cancer Prevention and Control Federation, and an anonymous online survey was adopted. The first part of the questionnaire was informed consent. Respondents participated voluntarily, filled in all questions, and submitted them, which was regarded as a valid questionnaire without any compensation. This study was conducted in accordance with the principles of the Declaration of Helsinki and was approved by the Hospital Ethics Committee with ethics review number 2022ky090.

### Survey tools

(1) General information questionnaire, with 10 items including gender, age, education level, professional title, working years, working department, and whether training on bone health is undertaken in the context of cancer and treatment

(2) Questionnaire on knowledge of oncology nurses about skeletal-related events: The questionnaire was compiled by reading domestic and foreign literature [13, 17–23], interviewing with oncology nurses, and consulting with experts on the topic, including three parts: nurses’ role and job responsibilities, knowledge of SREs and CTIBL, and understanding of and confidence in skeletal health management, for a total of 23 topics. The content validity index of the questionnaire is 0.95, the content validity index of each item is 0.67–1.00, the degree of enthusiasm of experts is 100%, the degree of authority of expert opinions is 0.90, and the degree of coordination of expert opinions is 0.12.

### Data collection and quality control methods

This study adopted a cross-sectional design, questionnaires were distributed to cancer nurses by the Hebei Cancer Prevention and Control Federation, and an anonymous online survey was adopted. A total of 602 questionnaires were issued and valid, and the effective rate was 100%. Quality control in the following aspects:

(1) Preinvestigation: Before the formal investigation, 30 nurses in our hospital were selected to conduct a preinvestigation, and the questionnaire was revised and improved according to the feedback information from the preinvestigation.

(2) Selection and training of investigators: All investigators were oncology nurses with more than 5 years of work experience who had prior experience in conducting research and unified training.

(3) The questionnaire is presented and sent in the form of links, and all the questionnaire questions are set as mandatory items. If there is any omission, when you click “Submit,” a prompt will appear on the questionnaire page asking for completion information to ensure the completeness of the questionnaire filling.

## Results

### General information of oncology nurses (see Table 1)

**Table 1:** General information of oncology nurses (*N*=602)

Item	Classification	Quantity	Percentage (%)/mean
Gender	Male Female	24 578	4.00 96.00
Age	20–59 years	602	33.80±6.99
Education level	Junior college Undergraduate Master	77 519 6	12.80 86.20 1.00
Professional title	Nurse Senior nurse Charge nurse Vice-director nurse Chief superintendent nurse	80 203 285 31 3	13.29 33.72 47.34 5.15 0.50
Working years	<1 year 1–<4 years 4–<8 years 8–<12 years 12–<15 years ≥15 years	81 87 90 160 67 117	13.46 14.45 14.95 26.58 11.13 19.44
Working department	Oncology Breast surgery Urology surgery Orthopedics Hospice care Radiotherapy Thoracic surgery Other	132 132 50 40 5 109 109 25	22.03 22.03 8.37 6.61 0.88 18.06 17.62 4.40
Whether they have received training on bone health	Yes No	228 374	37.80 62.20

### Role positioning and responsibilities of oncology nurses

**Fig. 1:**
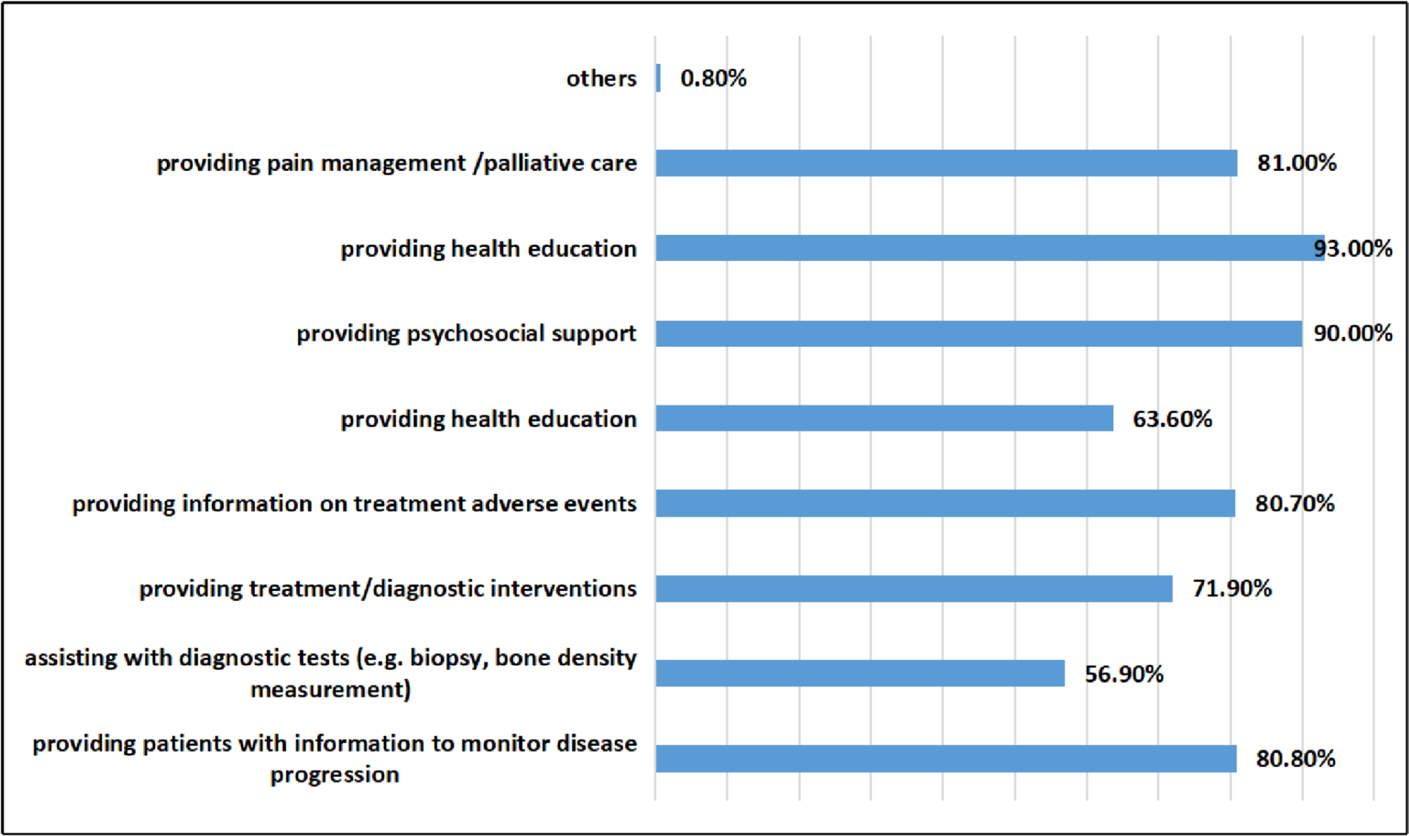
The survey results of oncology nurses on their own roles

**Fig. 2:**
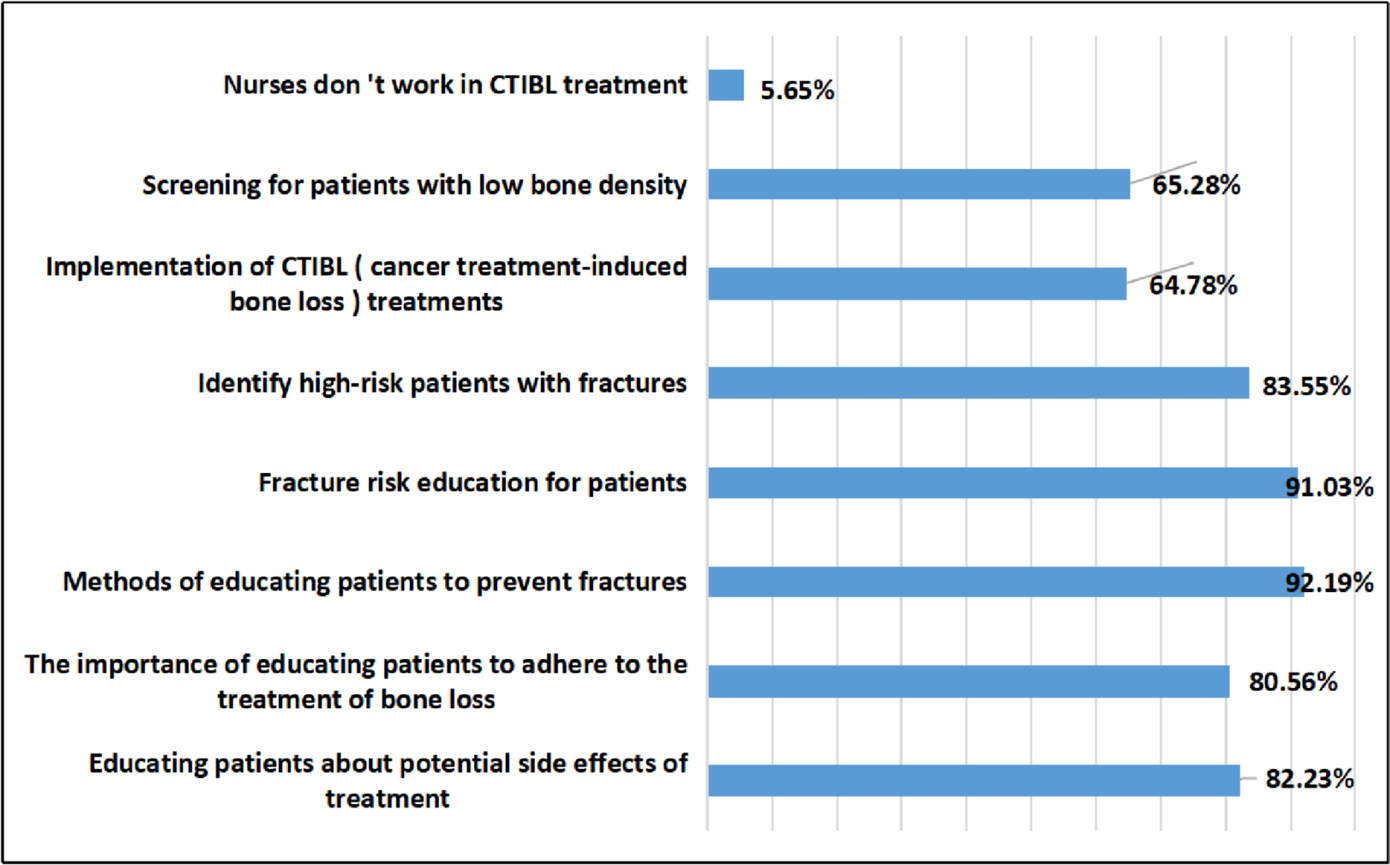
The survey results of oncology nurses’ understanding of their own responsibilities

In this survey, 37.80% of oncology nurses received training on bone health. Figures 1 and 2 display the survey results of oncology nurses’ understanding of their own roles and job responsibilities.

### Understanding of and confidence in managing bone health

#### Oncology nurses’ understanding of bone health management

**Fig. 3:**
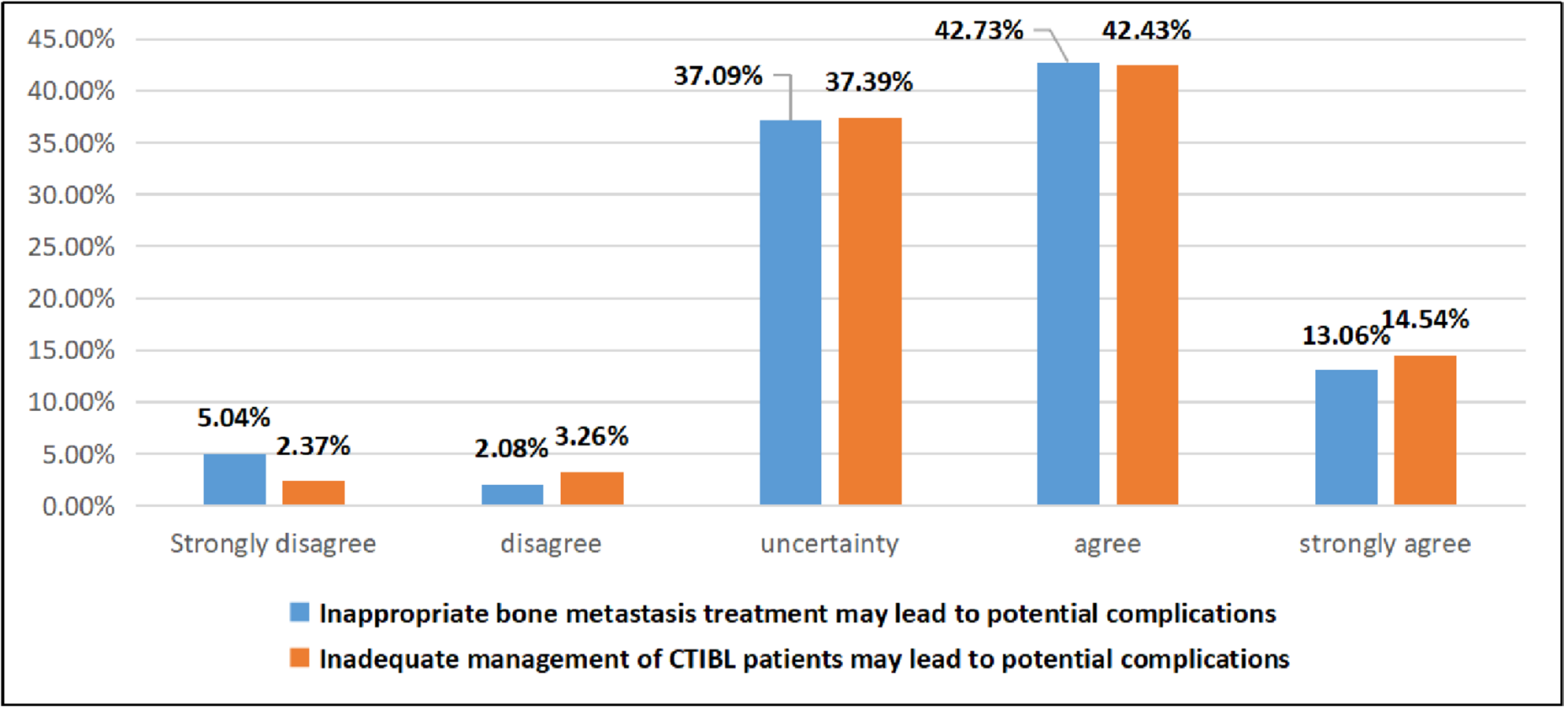
The survey results of oncology nurses’ understanding of managing bone health

The results of this study show that less than 50% of oncology nurses agree that inappropriate bone metastasis treatment measures and inadequate management will lead to potential complications. The specific survey results are shown in Fig. 3.

#### Oncology nurses’ confidence in managing patients with bone metastases

**Fig. 4:**
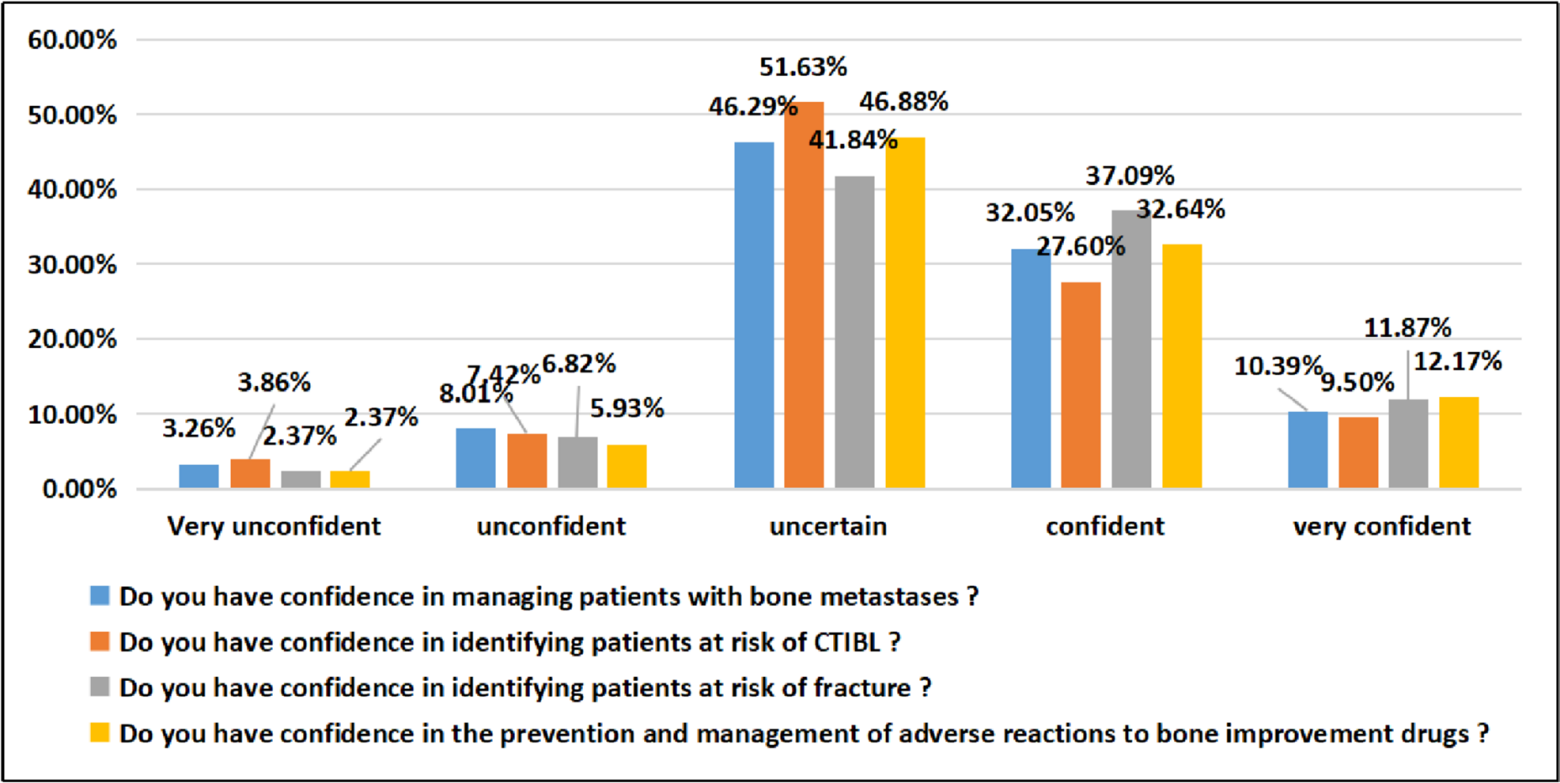
The survey results of oncology nurses’ confidence in managing patients with bone metastases

The results of this study show that approximately 50% of oncology nurses expressed uncertainty and lacked confidence in managing patients with bone metastases and in risk identification. The specific survey results are shown in Fig. 4.

### Survey on knowledge awareness of bone-related events

#### Oncology nurses’ awareness of bone loss prevention measures

**Fig. 5:**
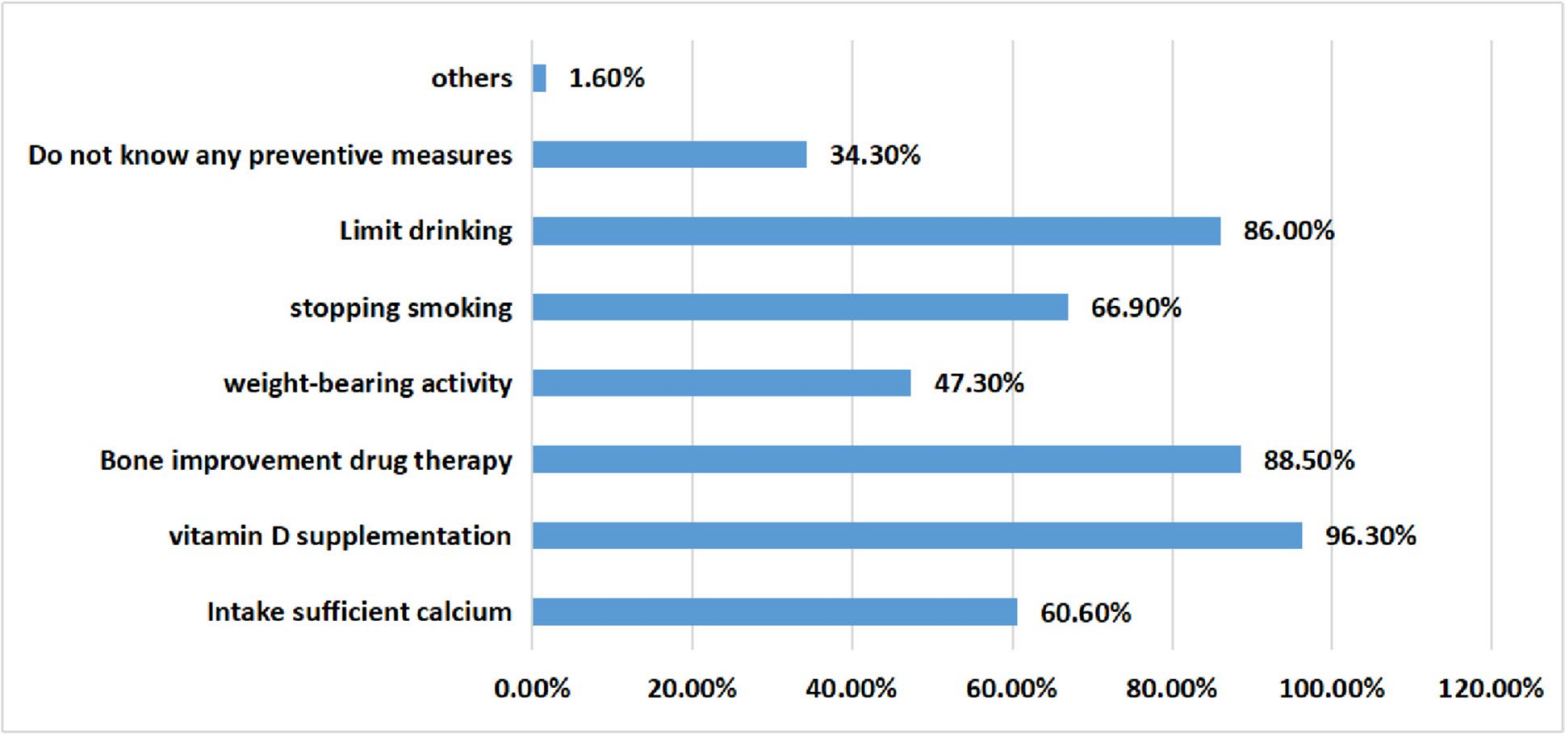
The survey results of awareness of bone loss prevention measures

A total of 96.30% and 88.50% of oncology nurses believed that supplementing vitamin D and applying bone-improving drugs could prevent bone loss, 86.00% believed that limiting alcohol could prevent bone loss, 60.60% believed that adequate calcium intake could prevent bone loss, only 47.30% believed that weight-bearing exercise can prevent bone loss, and 34.30% of oncology nurses did not know any preventive measures. The specific survey results are shown in Fig. 5.

#### Oncology nurses’ awareness of risk factors for pathological fractures

**Fig. 6:**
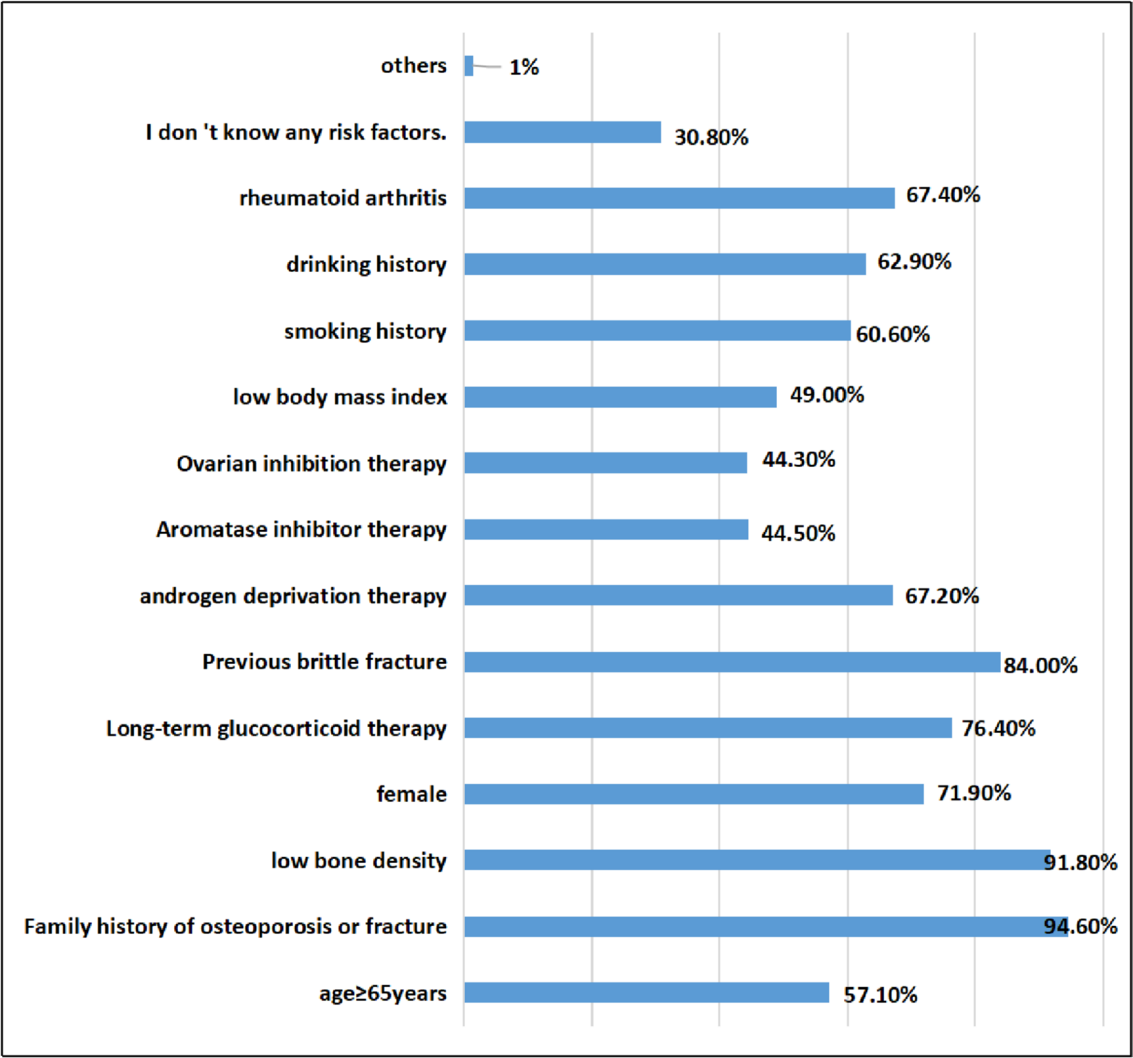
The survey results of awareness of pathological fracture risk factors

A total of 94.60%, 91.80%, and 84% of oncology nurses believed that osteoporosis or family history of hip fracture, low bone density, and previous fragility fractures were risk factors, and 76.40%, 71.90%, and 67.40% of oncology nurses believed that long-term glucocorticoid treatment, female sex, and rheumatoid arthritis were risk factors. A total of 67.20%, 62.90%, and 60.60% of oncology nurses believed that androgen deprivation therapy, drinking history, and smoking history were risk factors, less than 50% of oncology nurses believed that aromatase inhibitor therapy, ovarian suppression therapy, and low body mass index were risk factors, and 30.8% did not know any risk factors. The specific survey results are shown in Fig. 6.

### Oncology nurses’ barriers to recognizing and managing skeletal-related events

#### Recognizing barriers to bone loss prevention and risk factors

**Fig. 7:**
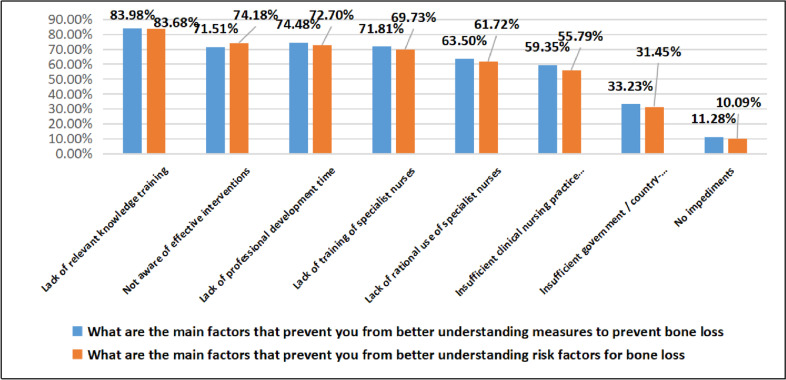
Findings of awareness of barriers to bone loss prevention and risk factors

The main reasons hindering oncology nurses from understanding bone loss prevention and risk factors are the lack of relevant knowledge training, lack of understanding of effective intervention measures, and lack of professional development time and training of specialized nurses, followed by the lack of rational use of specialized nurses, insufficient clinical nursing practice guidelines, and country/government-specific guidelines, and only approximately 10% of oncology nurses believe that there are no impediments. The specific survey results are shown in Fig. 7.

#### Barriers to the use of bone-modifying drugs as an intervention to support bone health

**Fig. 8:**
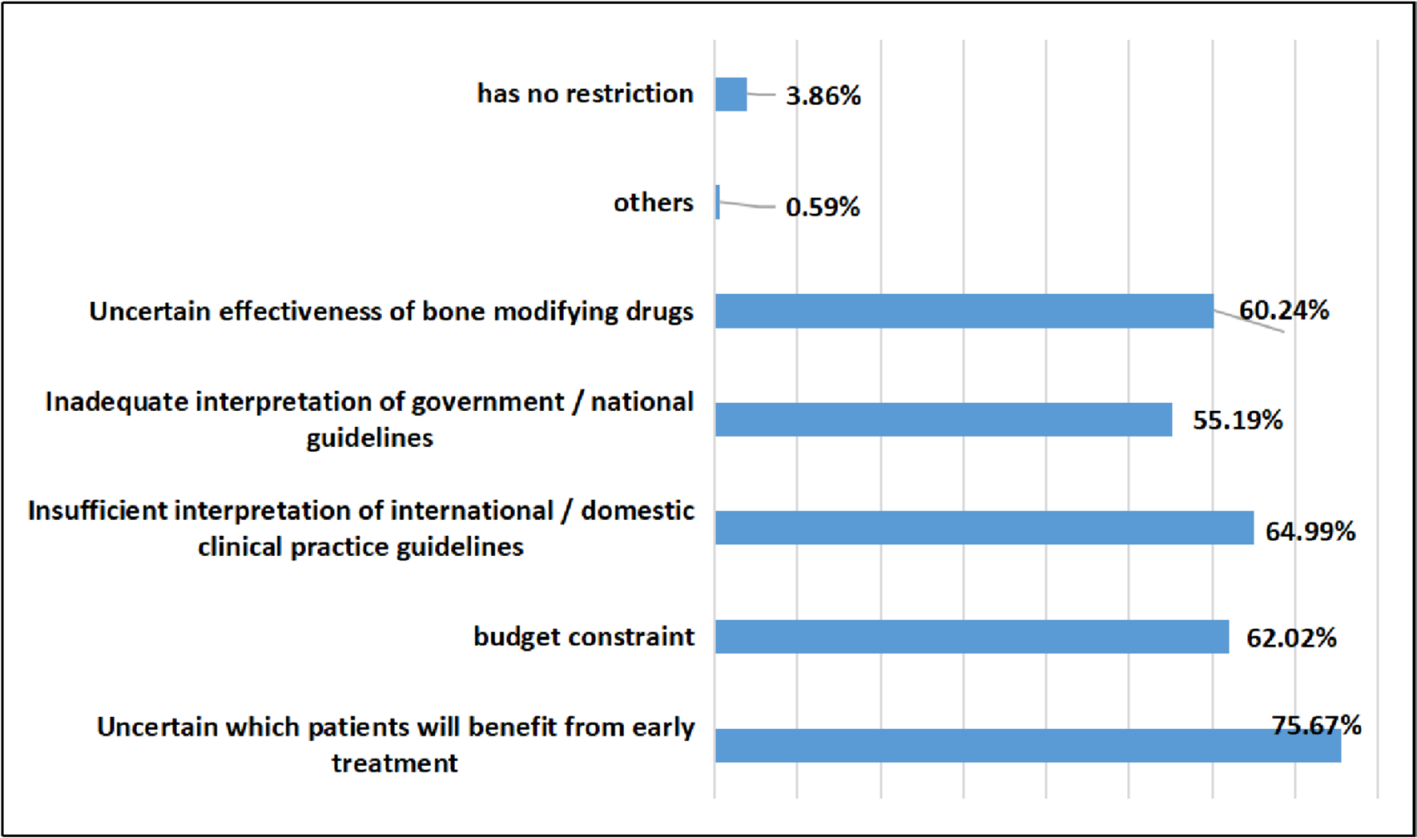
Findings of barriers to the use of bone-modifying drugs as an intervention to support bone health

A total of 75.67% of oncology nurses thought it was uncertain which patients would benefit from early treatment, 62.02% thought it was budget constraints, 64.99% thought it was insufficient interpretation of clinical practice guidelines, 60.24% thought there was uncertainty about the effectiveness of bone-modifying drugs, 55.19% thought that the interpretation of national/government guidelines was insufficient, and 3.86% thought that there was no limit. The specific survey results are shown in Fig. 8.

### The main obstacles for oncology nurses to optimize managing patients with bone metastases

**Fig. 9:**
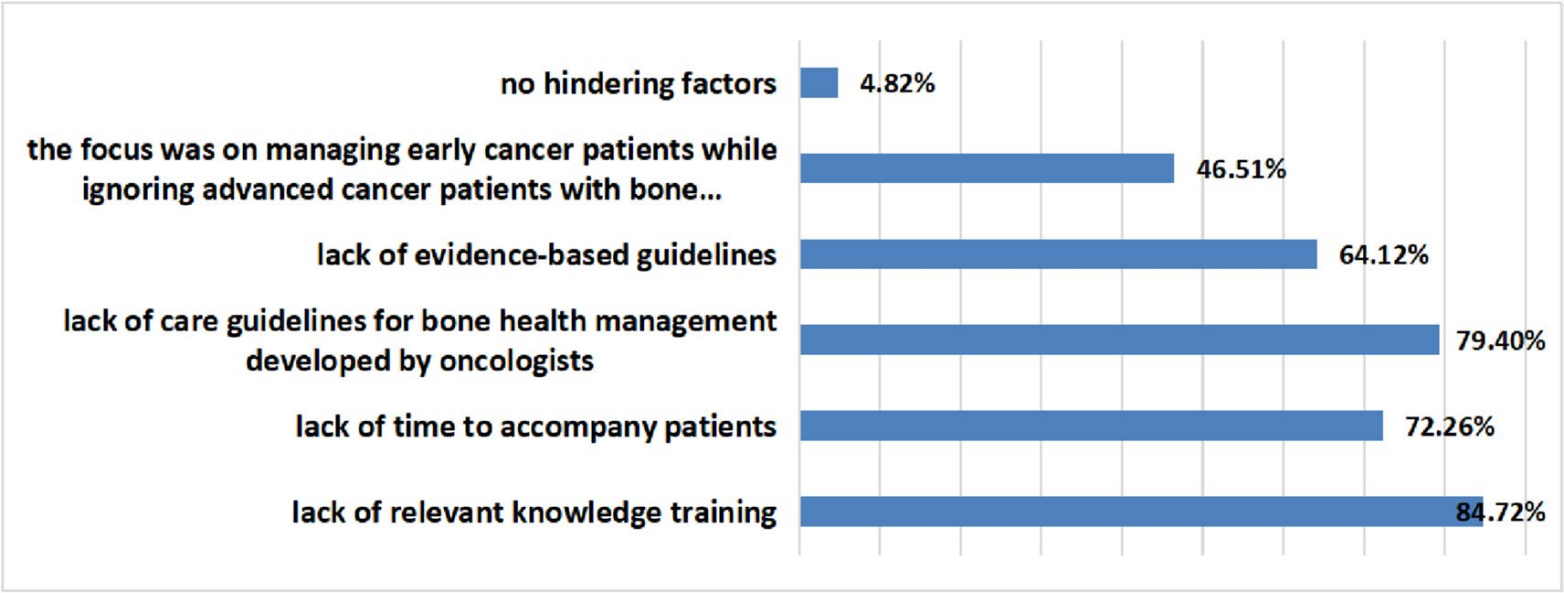
The survey results of the main obstacles in the optimal management of patients with bone metastases by oncology nurses

A total of 84.72% of oncology nurses believed that the lack of relevant knowledge training was the main obstacle, 72.26% believed that the main factor was a lack of time to accompany patients, 79.40% believed that it is the lack of care guidelines for bone health management developed by oncologists, 64.12% believed that there was a lack of evidence-based guidelines, 46.51% thought that the focus was on managing early cancer patients while ignoring advanced cancer patients with bone metastases, and 4.82% thought there were no hindering factors. The specific survey results are shown in Fig. 9.

## Discussion

### Insufficient knowledge of oncology nurses in managing the bone health of cancer patients

With the increasing survival rate of cancer patients and the increasing demand for health care, the training of oncology nurses has received increasing attention at home and abroad [22]. There are problems such as poor pertinence, unclear goals, and incomplete training content in the training of oncology nurses in China, and oncology nurses have not been given the opportunity to fully embrace their advantages and roles in clinical nursing [24–26]. Through quantitative analysis, this survey found that oncology nurses have insufficient knowledge in managing the bone health of cancer patients, which needs to be further improved.

### Insufficient understanding of the role of oncology nurses

Oncology nurses are nursing experts in the field of cancer and play important roles [27] that are diverse [19, 20, 28], including providing patients with information to monitor disease progression, assisting with diagnostic tests (e.g., biopsy, bone density measurement), providing information on treatment adverse events, providing treatment/diagnostic interventions, providing psychosocial support, providing health education, and providing pain management/palliative care. This study found that oncology nurses do not have a comprehensive understanding of their roles, and only 56.90% and 63.60% of oncology nurses did not realize the importance of biopsy and bone mineral density measurement for bone health. In clinical care, oncology nurses have prolonged contact with patients and are more likely to establish effective communication than other medical staff, supporting and coordinating the multidisciplinary management team for bone health [23]. Sussman [20] confirmed that patients benefited from oncology nurse supportive care, including disease awareness, self-monitoring, lifestyle, functional exercise, and nutritional support, especially educating patients about bone-improving drugs such as bisphosphonates and the importance of denosumab administration to improve patients’ medication compliance. Monitoring the adverse reactions of patients with bone-improving drugs and taking appropriate preventive measures can improve the bone health outcomes of patients and reduce or delay the occurrence of bone-related events [13, 21, 29].

### Knowledge deficits in bone loss prevention measures

The causes of bone loss in cancer patients are as follows [30–32]: bone destruction caused by bone metastasis of advanced cancer; chemotherapy patients treated with cytotoxic drugs can directly lead to bone loss through cytotoxicity; radiotherapy and hormone therapy lead to bone loss. The above reasons can lead to the destruction of bone structural integrity in patients with bone metastasis of solid tumors, and SREs and CTIBL eventually occur, which is harmful to bone health, seriously affects the quality of life of patients, and has a considerable economic burden for patients [33]. Therefore, oncology nurses should pay attention to bone loss in cancer patients, assess the risk of bone loss, and take appropriate preventive measures [34]. The Practical Guidelines for Bisphosphonates in the Treatment of Osteoporosis [35] and the National Comprehensive Cancer Network Expert Panel on Bone Health in Cancer Patients [36] recommend calcium and vitamin D supplementation, smoking cessation, alcohol restriction, weight-bearing or resistance training, and application of bone-modifying drugs for patients at risk of bone loss. During weight-bearing or resistance training, the force can be transmitted through the bones and converted into recognizable mechanical signals and cause a series of biochemical reactions to increase bone deposition, thereby increasing bone density and improving osteoporosis in patients [37, 38]. Multiple studies [30, 33, 34, 39] have shown that bone-modifying drugs such as bisphosphonates and denosumab are effective drugs to prevent and treat SREs and CTIBL. This study shows that oncology nurses have insufficient awareness of weight-bearing or resistance training and bone-modifying drugs to prevent bone loss and maintain bone health. Therefore, knowledge of weight-bearing training and bone-modifying drugs should be added to the bone health training plan of oncology nurses.

### Knowledge deficits in risk factors for pathological fractures

The International Clinical Practice Guidelines for Osteoporosis [40] noted that age over 65 years old, family history of osteoporosis or fracture, low bone mineral density, female sex, previous fragility fractures, low body mass index, and history of smoking and drinking are all high-risk factors for pathological fractures. The standard of adjuvant endocrine therapy for breast cancer in premenopausal women [41] recommends ovarian suppression therapy tamoxifen or aromatase inhibitors and androgen deprivation therapy (ADT) for prostate cancer patients with antiandrogen drugs. Castration treatment [42], long-term use of glucocorticoids for rheumatoid arthritis [43], and the application of the above drugs can lead to bone loss, adverse effects on bone health, and even pathological fractures [31]. The results of this study showed that only approximately 50% of oncology nurses were aware that aromatase inhibitor therapy, ovarian suppression therapy, androgen deprivation therapy, and low body mass index were risk factors for pathological fractures. More than half of oncology nurses did not use or did not know how to use guidelines. Therefore, strengthening the training of oncology nurses on cancer treatment methods and frontier medical knowledge, guiding them to actively consult domestic and foreign practice guidelines, and guiding oncology nursing work through evidence are necessary.

### Knowledge defects in health education

Cockle’s [19] research confirmed that cancer patients can benefit from the health education of oncology nurses, especially the implementation of targeted health education and assessing cancer patients with bone metastases, and CTIBL can improve the use of bone-improving drugs such as bisphosphonic acid. Adherence to salt and denosumab improves bone health outcomes in cancer patients. However, this study showed that less than 50% of oncology nurses were aware of the need to educate patients with low bone mineral density for regular screening and to educate patients at high risk of fracture for early treatment of CTIBL. Oncology nurses have little knowledge of bone health preservation strategies, screening tests, and the impact of cancer treatment on bone health [13]. Therefore, managers of medical institutions should focus on the above knowledge deficits in health education, and strengthen the training of bone health screening strategies and CTIBL knowledge for oncology nurses.

### Oncology nurses have insufficient confidence in the bone health management of cancer patients

This study found that only approximately one-third of oncology nurses are confident in managing patients with bone metastases, identifying patients at risk of CTIBL and pathological fractures, and preventing and managing side effects of bone-modifying drugs, which is consistent with Drudge’s [13] study, and the reason for the analysis is related to the lack of knowledge of bone health and the role of oncology nurses. In this study, there were bone health knowledge deficiencies, especially in bone loss prevention measures and pathological fracture risk factors. Managers should pay attention to the difficulties encountered by oncology nurses in nursing practice, and formulate standardized nursing plans for bone health of patients with advanced cancer, which is helpful to improve the management ability of oncology nurses for bone metastasis and CTIBL patients, and enhance their self-confidence.

### Obstacles to maintaining bone health

Prevention measures for bone loss and awareness of risk factors for pathological fractures were hindered by a lack of relevant knowledge training, unfamiliarity of effective intervention measures, and lack of professional development time and training of specialized nurses. The main obstacles to optimal management of patients with bone metastases and CTIBL patients are lack of relevant knowledge and training, lack of time to accompany patients, and lack of care guidelines for bone health management developed by oncologists. The main factors that hinder patients from receiving bone modification drugs are lack of knowledge and lack of bone modification drugs. Turner [14] reported a nurse-led bone health support clinic for cancer patients at a university medical center in London, which provided continuous care for cancer patients with SREs and CTIBL or patients at risk of bone loss. The study assessed and managed the patients’ bone health in a timely manner and highlighted the value of oncology nurses. Patients were satisfied with the support and education provided by nurses.

In summary, in spite of this, osteolytic lesions related to multiple myeloma (MM) should not be ignored, and the proportion of newly diagnosed MM patients with osteolytic lesions and SREs was as high as 80% [44]. Consider the atypia of some MM bone lesions (which can be manifested as bone masses and IGD-type MM patients with osteosclerosis accompanied by POEMS, namely polyneuropathy, organomegaly, endocrinopathy, and monoclonal immunoglobulin) and skin change syndrome) and MM-related complications (hypercalcemia, kidney damage, infection, etc.) [45]. Oncology nurses in the department of hematology need different knowledge from those in the department of solid oncology to manage bone health in patients with MM, so the two groups of people were studied separately. Research on the level of knowledge of oncology nurses in hematology on bone health of MM patients is ongoing.

## Conclusion

The bone health of cancer patients has received increasing attention. Maintaining bone health plays a key role in comprehensive cancer treatment. Bone health management requires the collaboration of a multidisciplinary team including oncology nurses. Oncology nurses must have a full understanding of bone health, have keen observation skills and accurate assessment skills, and master the knowledge of comprehensive cancer treatment, including bone-modifying drugs, surgery, radiotherapy, and endocrine therapy. Serious complications such as calcemia and spinal cord compression should be promptly noted, evaluated, and intervened, and targeted health education should be provided.

To improve the self-confidence of oncology nurses in managing SREs and CTIBL patients, continuously improving the training system of oncology nurses and enriching the bone health training content of cancer patients are necessary. The government and the competent departments of health institutions should provide financial and career development support for the obstacles to maintaining bone health, formulate guidelines for the practice of bone health care for cancer patients, promote the establishment of bone health support clinics led by oncology nurses, give attention to the role of oncology nurses in the bone health management of cancer patients, and improve their bone health outcomes.
